# Prevalence and Risk Factors of Prediabetes in Young Saudi Females in a University Setting

**DOI:** 10.4314/ejhs.v30i6.11

**Published:** 2020-11

**Authors:** Rabia Latif, Nazish Rafique

**Affiliations:** 1 Department of Physiology, College of Medicine, Imam Abdulrahman Bin Faisal University

**Keywords:** Prediabetic state, Risk factors, Adolescence, Young

## Abstract

**Background:**

Studies reporting prediabetes prevalence in young Saudis are almost a decade old. The present study determined prediabetes prevalence and its associated risk factors (body composition, lipid profile, blood pressure and physical activity) in young Saudi females.

**Methods:**

Three hundred Saudi females (18–20 years old), studying in year 2 and 3 at Imam Abdulrahman Bin Faisal University, were categorized as normoglycemic or prediabetic based on fasting plasma glucose criteria of World Health Organization (WHO) and American Diabetes Association (ADA). Anthropometric measurements, lipid profile, atherogenic indices, and physical activity data were compared. Association between fasting blood glucose and study variables was found by Bivariate analysis (Spearman Correlation for non-parametric variables and Pearson correlation for parametric) followed by Binary Logistic Regression analysis.

**Results:**

Prevalence of prediabetes by WHO and ADA criteria were 11.3% and 18.7% respectively. Systolic, diastolic and mean arterial pressures, waist circumference, waist-hip and waist-stature-ratios were significantly raised in prediabetics compared to normoglycemic (WHO criteria p-values; 0.03, 0.003, 0.005, 0.01, 0.01, 0.04 respectively; ADA criteria 0.04, 0.001, 0.02, 0.02, 0.03, 0.01 respectively). For each unit increase in systolic, diastolic and mean arterial pressures, and waist circumference, the odds of becoming prediabetic increased by a factor of 1.02, 1.05, 1.04 and 1.03 respectively by WHO criteria: and 1.01, 1.03, 1.02, and 1.02 respectively by ADA criteria

**Conclusion:**

Substantial numbers of young females in our university are suffering from prediabetes. An increase in systolic, diastolic, and mean arterial pressures and waist circumference are significant risk factors for prediabetes in young females.

## Introduction

Prediabetes (a condition of altered glucose homeostasis), is associated with a high risk of developing type 2 diabetes (1). A rise in prediabetes prevalence has been reported in the general population of a) China, where prediabetes has risen from 7.3% in 2000–2001 (2) to 15.5 % in 2010 (3); b) UK, from 11.6% in 2003 to 35.3% in 2011 (4); c) India, from 14.2% in 2001 to 25% in 2018 (5); d) US, from 10.2% in (1988–1994) to 18.5% in (1999–2012) (6). All these studies point towards the trend of a substantial increase in the percentage of prediabetes internationally. The same trend has been observed in adolescents worldwide, regardless of race. In a recent report from the US, the prevalence of prediabetes in adolescents increased from 9% in 1999–2000 to 23% in 2007–2008 (7). Likewise, in Canada, the prevalence of prediabetes increased from 1% (8) to 10% (9) in adolescents.

In Middle East countries, lifestyle changed significantly after the discovery of oil in 1935. The healthier traditional diet of dates, wheat, camel milk, fruits, vegetables and seafood was replaced by processed foods rich in sugar, salt, and oil imported from the west. These unhealthy eating habits and sedentary lifestyles led to a marked increase in obesity and diabetes (10). In fact, the Middle East and North Africa (MENA) have the second-highest rate of diabetes surge worldwide, where the prevalence of diabetes and prediabetes is predicted to rise by 96% and 82 % respectively by 2045. This will result in an increase of 55% in terms of cost of diabetic treatment, management of complications, disability and loss of productivity in MENA countries (11).

A recent review on the prevalence of prediabetes in the MENA region has concluded that the true prevalence of prediabetes has not been adequately determined across this region (12). KSA is one of those few countries in the MENA region which has the maximum prevalence (>12%) of diabetes (20–79 years) (11). In KSA, studies are available documenting epidemiological trends for type 2 diabetes (13–15). There are, however, insufficient data documenting trends of prediabetes, especially in adolescents. One of the studies determined impaired fasting glucose in subjects aged ≥ 30 years in the year 2004–2005 and reported it as 18.2% (14). Regarding prediabetes prevalence in adolescents, only two studies have been conducted so far (13,15). These studies have reported prevalence based on data that is about a decade old. Al-Rubeaan (13) and Al-Daghri et al. (15) collected data in 2007–2009 and 2010, respectively. A fresh study is needed to evaluate the status of the prediabetes prevalence in the Saudi population and the fruitfulness of the efforts of the Ministry of Health.

To address this gap in knowledge, we performed a single-center cross-sectional study with the following study aims: 1) to determine the prevalence of prediabetes in the Saudi adolescents 2) to determine differences in key clinical parameters between adolescents having prediabetes, or normal glucose metabolism, and 3) to assess the factors associated with prediabetes in adolescents; using two different diagnostic criteria a: World Health Organization (WHO) and b: American Diabetes Association (ADA). We also examined heterogeneity in the prevalence and determinants of prediabetes across these two criteria.

Keeping in view the universal trend of an upward drift in the prevalence of prediabetes, we hypothesized a prevalence of more than 6.12% and 5.6% (13,15) in our study subjects. Since abdominal obesity, increased body fat %, hypertension, hypertriglyceridemia and low levels of low-density lipoprotein-cholesterol can lead to a fivefold increased risk for the development of diabetes (16), we hypothesized a significant association of these risk factors with fasting blood glucose in our subjects. Because of strict gender segregation policy observed in institutions all over the kingdom, the study was conducted in a female campus, and all study participants were females only.

## Subjects and Methods

**Subjects**: Our subjects were Saudi female students (18–20 years), studying in year 2 and 3 at various colleges of Imam Abdulrahman Bin Faisal University. Ethical approval for this cross-sectional study was obtained from the Institutional Review Board of the university (IRB-2014-01-169). All experiments complied with the World Medical Association Declaration of Helsinki regarding the ethical conduct of research involving human subjects. To attract the students, small talks were given by the researchers in each class highlighting the problems caused by diabetes and its complications and benefits of an early diagnosis. Our inclusion criteria was 18–20 years old Saudi female students registered in our university, willing to participate in the study and not using any regular medication for the last three months. The students who were pregnant, lactating or suffering from any diagnosed chronic illness were excluded from the study.

**Sample size calculation**: The sample size was calculated using single population formula considering level of confidence as 95%, value of Z corresponding to that was 1.96; P = 25 % (obtained from a pilot study conducted by the researchers); d (relative precision) as 20 % of P i.e. 20 /100 * 25 = 5. By putting the values, n = 1.96^2^*25 (75)/5^2^, a sample size of 300 was finalized. A total of 300 young females participated in the study and provided verbal informed consent.

**Anthropometric measurements**: To have an exact idea about the body composition, three main parameters including Body Mass Index (BMI), Waist Circumference (WC) and Skinfold thickness were used. BMI is limited in distinguishing between fat mass and muscle mass. WC, though it provides high sensitivity and specificity for central fat evaluation, it gives a general overview of body composition only (18). An inexpensive method which overcomes WC and BMI limitations is measurement of body density and body fat percentage by evaluating skinfold thickness. Skinfold thickness techniques provide a correct assessment of children's and adolescents' body composition (19).

BMI was calculated as the subjects' body mass divided by the square of the height (kg/m2). WC was measured midway between the lowest rib and the iliac crest after a normal exhale. Hip circumference was measured around the broadest portion of the buttocks.

Skinfold thicknesses from four different anatomical sites around the body (1) triceps: halfway between the acromion process and the olecranon process; 2) Biceps: at the same level as the triceps skinfold; 3) Subscapular, about 20 mm below the tip of the scapula, at an angle of 45′ to the lateral side of the body; 4) Suprailiac, about 20 mm above the iliac crest, in the axillary line were measured to the nearest 0.1 mm with skinfold calipers.

To calculate body density, the log of the sum of four skinfold sites (triceps, biceps, subscapular and suprailiac) was substituted into the equation of Durnin and Womersley (19). D = 1.1599 – (0.0717*L) Where D = predicted density of the body (g/ml), and L = log of the total of the 4 skinfolds. The density value thus obtained was then converted to percent body fat (%) with Siri Equation (20); % Body Fat = (495 / Body Density) – 450.

**Blood pressure measurements**: Blood Pressure (BP) was recorded by Welch Allyn Spot Vital Signs monitor using a proper blood pressure cuff size wrapped around the arm at heart level. Pulse pressure and mean arterial pressure were calculated by the formulae Systolic BP-Diastolic BP and Diastolic BP + 1/3 of pulse pressure, respectively.

**Biochemical measurements**: Portable devices like Accu-Chek Performa and Accutrend® Plus system (Roche Diagnostics) were used to determine fasting plasma glucose (FPG) and lipid profiles, respectively. Fasting of at least 8 hours was recommended for blood glucose and 10–12 hours for lipid profile (High-density lipoproteins (HDL), Low-density lipoproteins (LDL), triglycerides and total cholesterol). For glucose measurement, a glucose test strip was inserted into the meter. A blood drop taken from the fingertip was applied on the top of the test strip in a proper window, and the result was displayed on the screen. For the lipid profile, the blood drop was placed on the lipid profile test strips which had special chemicals that changed color after a few minutes. The final color was matched against a color guide that was included with the kit and the information about the HDL, LDL, triglycerides and total cholesterol in the droplet of blood was obtained. Following this, atherogenic indices were calculated (21):

Castelli's Risk Index-I (CRI-I) = Total cholesterol/HDL

Atherogenic Index (AI) = LDL/HDL

Atherogenic Coefficient (AC) = (Total cholesterol-HDL)/HDL

Surrogate marker of Insulin resistance = Triglycerides/HDL

**Physical activity status**: Physical activity data were gathered through the International Physical Activity Questionnaire-short form (IPAQ-SF) (22). Responses were converted to Metabolic Equivalent (MET) minutes/week and then total physical activity MET-minutes/week were obtained by taking sum of walking, moderate and vigorous MET-minutes/week scores (23).

**Prediabetes diagnostic criteria**: Cutoff points of fasting plasma glucose for the diagnosis of prediabetes were set at 110–125 mg/dl and 100–125 mg/dl as per the criteria of WHO (24) and ADA (25) respectively.

**Statistical analyses**: Distribution of data were checked by the Shapiro Wilk Test. Normally distributed variables (lipid profile) and nonnormally distributed variables (all remaining variables) were compared across the two categories of glycemic status by parametric (independent sample t-test) and non-parametric tests (Mann Whitney U test) respectively. Associations between fasting blood glucose and each of the variables were found by bivariate analysis (Spearman Correlation for nonparametric variables and Pearson correlation for parametric) first followed by Binary Logistic Regression analysis to understand the effects of variables. In Binary Logistic Regression analysis, the presence or absence of prediabetes was taken as a dependent variable and the rest of all variables as covariates. All tests were two-sided with a significance level set at *P*<0.05.

## Results

Out of a total student population of 3000, 300 students participated in this study (participation rate = 10%). Prevalence of the prediabetes was 11.33% and 18.67% by WHO and ADA criteria, respectively in our study participants (Table 1). Among pressure measurements, systolic, diastolic and mean arterial pressure, and in anthropometry, waist circumference, waist-hipratio, and waist-stature-ratio were found to be significantly increased in prediabetics by both criteria, WHO and ADA (Table 1).

**Table 1:** Characteristics of students with normoglycemia and prediabetes (WHO and ADA criteria)

Parameters	WHO criteria (FPG 110–125 mg/dl)			ADA criteria (FPG 100–125 mg/dl)		
	
	Normoglycemia	Prediabetes	P value	Normoglycemia	Prediabetes	P value
Number (%)	263 (87.67)	34 (11.3)	---	241 (80.33)	56 (18.7)	---
Age (years)	18.8±2.3	19.2±1.5	0.35	18.4±1.3	18.93±1.7	0.54
Fasting Blood Sugar (mg/dl)	86.14±8.54	113.91±5.04	0.02	79.29±10.18	110.78±12.7	0.01
Pressure measurements						
Systolic Blood Pressure (mmHg)	112.70±11.84	116.20±12.2	0.03	106.33±8.99	113.34±12.6	0.04
Diastolic Blood Pressure (mmHg)	70.38±7.96	73.42±6.71	0.003	70.66±3.99	72.84±2.23	0.001
Pulse Pressure (mmHg)	42.32±8.27	42.78±8.51	0.52	36.76±6.54	41.34±5.35	0.87
Mean Arterial Pressure (mmHg)	84.49±8.59	87.68±7.98	0.005	83.77±6.12	85.88±7.36	0.02
Anthropometry						
Body mass (kg)	57.89±15.45	60.44±16.03	0.23	54.89±10.44	58.55±12.76	0.31
Height (cm)	156.81±8.24	157.44±6.70	0.79	157.83±8.99	158.98±8.72	0.44
Body Mass Index	24.19±14.81	24.01±6.85	0.38	22.78±5.32	23.4±4.11	0.56
Waist Circumference (cm)	71.76±11.04	75.92±11.77	0.01	67.82±7.45	73.88±10.23	0.02
Hip Circumference (cm)	98.43±13.04	100.86±11.9	0.19	96.54±10.74	98.74±8.99	0.12
Waist-Hip-Ratio	0.73±0.07	0.75±0.06	0.01	0.71±0.03	0.75±0.05	0.03
Waist-Stature-Ratio	0.46±0.07	0.47±0.10	0.04	0.43±0.19	0.47±0.23	0.01
Body density (g/ml)	1.02±0.10	1.01±0.13	0.29	1.01±0.30	1.02±0.21	0.37
% Body fat	31.12±6.80	31.67±7.11	0.36	31.88±5.87	32.93±7.65	0.55
Lipid profile						
High density lipoprotein (mg/dl)	60.27±19.22	55.14±15.82	0.25	58.63±16.88	53.54±13.18	0.48
Low density lipoprotein (mg/dl)	72.82±20.03	80.38±21.43	0.16	77.13±18.65	85.22±17.52	0.43
Triglycerides (mg/dl)	86.82±32.29	93.14±42.97	0.50	88.15±24.37	91.23±35.84	0.26
Total Cholesterol (mg/dl)	151.86±53.85	157.52±24.6	0.65	146.73±44.73	152.35±29.1	0.72
Atherogenic Indices						
Total Cholesterol/High density lipoprotein	2.16±0.87	2.81±0.38	0.63	2.53±1.13	2.86±0.98	0.16
Low density lipoprotein/High density lipoprotein	1.18±0.43	1.50±0.16	0.31	1.33±0.54	1.60±0.42	0.81
(Total Cholesterol-High density lipoprotein)/ High density lipoprotein	1.73±0.21	1.97±0.32	0.07	1.51±0.29	1.87±0.21	0.84
Triglycerides/ High density lipoprotein	1.50±0.27	1.71±0.56	0.24	1.51±0.33	1.71±0.17	0.75
Physical activity duration (minutes/day)						
Walking	26.74±6.53	22.87±8.44	0.54	31.55±10.21	27.35±9.56	0.88
Moderate intensity activities	12.33±3.21	10.54±7.8	0.32	15.62±7.58	13.62±6.17	0.19
Vigorous intensity activities	8.03±1.64	9.04±3.1	0.85	13.15±4.99	12.18±5.89	0.45
Physical activity MET-minutes/week						
Walking	441.29±54.91	405.78±49.4	0.64	457.44±46.73	435.32±56.1	0.32
Moderate intensity activities	98.64±13.56	84.32±9.85	0.13	105.25±15.22	93.88±12.71	0.57
Vigorous intensity activities	64.24±7.12	68.39±6.54	0.28	79.55±11.86	75.83±9.23	0.86
Combined total physical activity scores	603.98±51.23	557.86±43.6	0.34	642±74.11	605±65.89	0.16

Walking MET-minutes/week = 3.3*walking minutes*walking days

Moderate MET-minutes/week = 4.0*moderate-intensity activity minutes*moderate days

Vigorous MET-minutes/week = 8.0*vigorous-intensity activity minutes*vigorous-intensity days

Total physical activity MET-minutes/week = sum of Walking + Moderate + Vigorous MET minutes/week scores.

Abbreviations: WHO: World Health Organization; ADA: American Diabetes Association; MET: Metabolic equivalen

All four pressure measurements (systolic, diastolic, pulse and mean arterial pressure) and six of the anthropometric measurements (body mass, body mass index, waist and hip circumferences, waist-stature-ratio, % body fat) showed a significant direct relationship with fasting blood glucose (Table 2). However, the strength of the relationship was weak. Body density was the only variable that showed an inverse relationship, significant but weak.

**Table 2:** Pearson Correlation of Fasting blood sugar with study variables

Parameter	Spearman/Pearson Correlation	P value
Pressure measurements		
Systolic Blood Pressure (mmHg)	0.17	0.003
Diastolic Blood Pressure (mmHg)	0.15	0.01
Pulse Pressure (mmHg)	0.13	0.03
Mean Arterial Pressure (mmHg)	0.16	0.006
Anthropometry		
Body mass (kg)	0.21	0.000
Height (cm)	0.09	0.15
Body Mass Index	0.18	0.002
Waist Circumference (cm)	0.28	0.000
Hip Circumference (cm)	0.24	0.000
Waist-Hip-Ratio	0.12	0.05
Waist-Stature-Ratio	0.23	0.000
Body density (g/ml)	-0.17	0.005
% Body fat	0.16	0.008
Lipid Profile		
High density lipoprotein (mg/dl)	-0.15	0.21
Low density lipoprotein (mg/dl)	0.14	0.23
Triglycerides (mg/dl)	0.17	0.15
Total Cholesterol (mg/dl)	-0.04	0.74
Atherogenic Indices		
Total Cholesterol/High density lipoprotein	0.13	0.89
Low density lipoprotein/High density lipoprotein	0.08	0.12
(Total Cholesterol-High density lipoprotein)/ High density lipoprotein	0.17	0.83
Triglycerides/ High density lipoprotein	0.03	0.62
Physical activity duration (minutes/day)		
Walking	0.22	0.09
Moderate intensity activities	0.27	0.54
Vigorous intensity activities	0.15	0.18
Physical activity MET-minutes/week		
aWalking	0.22	0.08
bModerate intensity activities	0.17	0.52
cVigorous intensity activities	0.28	0.11
dCombined total physical activity scores	0.13	0.07

a Walking MET-minutes/week = 3.3*walking minutes*walking days

b Moderate MET-minutes/week = 4.0*moderate-intensity activity minutes*moderate days

c Vigorous MET-minutes/week = 8.0*vigorous-intensity activity minutes*vigorous-intensity days

d Total physical activity MET-minutes/week = sum of Walking + Moderate + Vigorous MET minutes/week scores.

In Binary Logistic Regression analysis of study participants categorized by WHO criteria, employing a .05 criterion of statistical significance, three of the pressure variables (systolic, diastolic and mean arterial pressure: odd ratios 1.02, 1.05 and 1.05 respectively), one of the anthropometric variables (waist circumference: odd ratio 1.03) had a significant effects (Table 3). These variables were found to have significant effects in study participants categorized by ADA criteria as well. Our results indicate that for each unit increase in systolic, diastolic and mean arterial pressures, and waist circumference, the odds of becoming prediabetic increased by a factor of 1.02, 1.05, 1.04 and 1.03 respectively by WHO criteria: and 1.01, 1.03, 1.02, and 1.02 respectively by ADA criteria.

**Table 3:** Binary logistic regression regarding presence or absence of prediabetes in normoglycemic and prediabetic females (WHO and ADA criteria)

Predictors	WHO Criteria		ADA Criteria	
	
	P value	Odds Ratio (or Exp (B))	P value	Odds Ratio (or Exp (B))
Pressure measurements				
Systolic Blood Pressure (mmHg)	0.04	1.02	0.03	1.01
Diastolic Blood Pressure (mmHg)	0.01	1.05	0.02	1.03
Pulse Pressure (mmHg)	0.70	1.01	0.65	1.12
Mean Arterial Pressure (mmHg)	0.01	1.04	0.02	1.02
Anthropometry				
Body mass (kg)	0.25	1.01	0.34	1.22
Height (cm)	0.58	1.01	0.25	1.45
Body Mass Index	0.92	1.0	0.33	1.2
Waist Circumference (cm)	0.01	1.03	0.04	1.02
Hip Circumference (cm)	0.18	1.01	0.21	1.43
Waist-Hip-Ratio	0.05	61.12	0.17	75.84
Waist-Stature-Ratio	0.18	10.85	0.09	15.32
Body density (g/ml)	0.61	0.55	0.44	0.85
% Body fat	0.58	1.01	0.31	1.91
Lipid Profile				
High density lipoprotein (mg/dl)	0.47	0.99	0.65	0.83
Low density lipoprotein (mg/dl)	0.28	1.02	0.43	1.16
Triglycerides (mg/dl)	0.88	0.999	0.62	0.77
Total Cholesterol (mg/dl)	0.78	0.998	0.54	0.843
Atherogenic Indices				
Total Cholesterol/High density lipoprotein	0.54	0.667	0.79	0.755
Low density lipoprotein/High density lipoprotein	0.36	0.234	0.45	0.54
(Total Cholesterol-High density lipoprotein)/ High density lipoprotein	0.12	1.002	0.76	1.23
Triglycerides/ High density lipoprotein	0.16	1.045	0.22	1.876
Physical activity duration (minutes/day)				
Walking	0.24	3.01	0.58	2.76
Moderate intensity activities	0.09	3.42	0.73	4.55
Vigorous intensity activities	0.11	2.87	0.31	1.54
Physical activity MET-minutes/week				
aWalking	0.28	0.092	0.34	0.176
bModerate intensity activities	0.35	0.065	0.56	0.187
cVigorous intensity activities	0.09	2.19	0.17	3.43
dCombined total physical activity scores	0.14	4.74	0.26	4.12

a Walking MET-minutes/week = 3.3*walking minutes*walking days

b Moderate MET-minutes/week = 4.0*moderate-intensity activity minutes*moderate days

c Vigorous MET-minutes/week = 8.0*vigorous-intensity activity minutes*vigorous-intensity days

d Total physical activity MET-minutes/week = sum of Walking + Moderate + Vigorous MET minutes/week scores.

Abbreviations: WHO: World Health Organization; ADA: American Diabetes Association; MET: Metabolic equivalent

## Discussion

Our study reported the prevalence of prediabetes to be 11.3% (WHO criteria) and 18.67% (ADA criteria) in Saudi girls (18–20 years old). As compared to the other countries in the Middle East, the prevalence found in our study is higher than the prevalence of prediabetes in the Qatari women (8.3%) (26) and lesser than the prevalence of prediabetes found in women of Iran (32.8%) (27), Oman (20.2 %) (28), and Kuwait (19.5 %) (29).

In comparison to the studies done locally involving adolescents, our study reports the highest prevalence of prediabetes so far. Previous studies conducted a decade ago have reported prevalence in Saudi adolescents as 6.12% and 5.6% (13,15). The reason for higher prevalence in our study as compared to previous studies could be an actual increase in the cases of prediabetes, or difference in methodology such as age, gender, residence (rural/urban) of the participants, etc. Al-Rubeaan (13) collected data from thirteen administrative regions of KSA (both rural and urban) from 2007–2009 and reported the prevalence of prediabetes as 6.12% among participants aged 6–18 years. Identification of risk factors by univariate and multivariate analysis revealed age ≥ 13 years and urban residency as a significant risk factor for diabetes and prediabetes in his study participants. As compared to Al-Rubeaan (13), all our subjects were 18–20 years old (late adolescent stage), gender female only, and resident of urban area. Keeping in mind that diabetes and prediabetes are diseases due to marked lifestyle changes and urbanization, recruiting subjects from an urban area only might be the underlying reason of the high prevalence rate of prediabetes in our study subjects. Likewise, Al-Daghri and his companions determined the prevalence of prediabetes in participants aged 7–17 years old as 5.6%, participants aged 8–45 years old as 10.9%, and overall prevalence in subjects aged 7–80 years as 9% (15). Though participants in the study by Al-Daghri (15) also belonged to an urban area (Riyadh region only), their age range of 8–45 years was much wider, and participants were older compared to the age range of our participants.

There was a good agreement in determinants/risk factors of prediabetes in both criteria. Prediabetic females by ADA or WHO criteria exhibited higher levels of cardiovascular risk factors such as systolic, diastolic and mean arterial pressures, WC, waist-hip and waist-stature ratios than normoglycemic females. The risk factors (blood pressure and waist circumference) that our study has identified to be associated with prediabetes in adolescents are the same conventional risk factors for insulin resistance/glucose metabolism identified earlier in adults (30). Bardenheier et al. (31) and Soriguer et al. (32) have reported an association of waist circumference with prediabetes in adults. Similarly, the association between hypertension and prediabetes has been reported previously (32–34). We did not find any association of lipid profile, atherogenic indices, body density and body fat % with the presence or absence of prediabetes, a finding in contrast to previous studies (19,35,36). The reason for this is unknown, but it might be speculated that since our subjects were very young (18–20 years of age), their disease/insulin resistance might be in the budding stage where it has not yet started affecting lipid metabolism. This needs to be confirmed in further studies. Physical activity parameters did not differ in normoglycemic and prediabetic subjects, and no association was found between physical activity parameters and presence/absence of prediabetes.

All our study participants who were diagnosed with impaired glucose metabolism i.e. diabetes and prediabetes were unaware of their state before. This shows that screening of prediabetes in this young age group might have been neglected in the country. According to the US Preventive Services Task Force (37), overweight and obese adults 40–70 years old should be screened for abnormal glucose levels. However, considering our results, we propose that the screening process should involve normal-weight adolescents as well. A focus on obese or overweight individuals may lead to missed opportunities for identification of hidden illness in healthy-weight individuals in developed countries. The rate of conversion of prediabetics into type 2 diabetes is 5–10%/year (38). Early detection of prediabetes (the predecessor of type 2 diabetes) at a young age, and taking steps to reduce its associated risk factors would reduce the incidence of diabetes and its financial burden on the families and the economy.

Our study concludes that a substantial number of female adolescents in our university are suffering from prediabetes. There is a good agreement in determinants/risk factors of prediabetes in WHO and ADA criteria. An increase in systolic, diastolic and mean arterial pressures and waist circumference are significant risk factors for prediabetes in female adolescents. The government should be more vigilant and take emergency measures to increase the awareness of prediabetes and its risk factors at all levels in society.

Our study has certain limitations. All our subjects belonged to a single university in a single region, and they were females only. Hence, our findings may not be generalized, and the current prevalence rate may not necessarily reflect the true prevalence at national level. We did not use an Oral Glucose Tolerance Test (OGTT) because it takes a longer time to complete. As our subjects were university students, they did not agree to come to the laboratory multiple times to complete OGTT, and thus some cases of diabetes and prediabetes might have been missed. Equations used for estimating body density and body fat % (Durnin and Womersley equation, Siri equation respectively) have not been cross-validated in the Saudi population. Last but not least, our study, being cross-sectional in nature, cannot determine the cause-effect relationship.

Our study indicates the need to promote early and targeted screening for prediabetes in Saudi adolescents. It is the utmost duty of the government policymakers to create such an environment in the country and within specific settings (school, university, home, workplace) that improves the health of every citizen. The Diabetes Prevention Program (DPP) in the USA (39), the Finnish Diabetes Prevention Study (DPS) (40) and the Chinese Da Qing Study (41) reveal that certain lifestyle modifications in dietary habits and physical activity pattern might have long term beneficial effects on glycemic control.
